# Patterns of interaction between patients, family members, and health professionals as markers of identity in palliative care

**DOI:** 10.1017/S1478951526103009

**Published:** 2026-06-22

**Authors:** Isabel Galriça Neto

**Affiliations:** Palliative Care Unit Hospital Luz-Lisboa, Lisbon University Medical School and Catolica Medical School, Lisboa, Portugal

## Theoretical framework: How to define PC identity

This theoretical–reflective work forms part of a broader research project examining the cultural and relational patterns that shape the identity of palliative care (PC) (Neto 2022, 2026). Its central aim is to advance the conceptualization of PC as a distinct clinical field, culturally situated and defined by a specific relational grammar.

PC represents one of the most profound transformations in contemporary medical thought and practice. Rather than a clinical specialty confined to disease-directed interventions, PC embodies a paradigmatic shift that relocates the center of gravity of clinical work “from disease to person, from cure to care, from quantity of life to its quality and meaning. Over several decades, building on the foundation contribuitions of Cicely Saunders - who introduced the concept of ‘total pain’ - alongside those of Balfour Mount and Eric Cassell, international consensus documents (Radbruch et al., 2020; WHO, 2020) have consolidated the conceptual fouddations of this field. The encompass a holistic and interdisciplinary model of care; a focus on quality of life and the relief of suffering in its broadest dimensions, as associated with advanced, life limiting illness; a strong ethical framework grounded in dignity, proportionality, and informed participation; and an explicit commitment to supporting families, including through bereavement care. These pillar elements delineate a clearly differentiated identity (Table 1), distinct from the acute-care biomedical model.

**Table 1.** Foundational values in PC Identity Table 1 long description.

Foundational values		
Category	Core element	Description
Constitutive	Holistic and interdisciplinary model	A holistic, interdisciplinary approach to care – one of the pillar elements consolidated by international consensus documents (WHO, Radbruck) as foundational to PC identity.
Constitutive	Quality of life and relief of suffering	Focus on quality of life and relief of suffering associated with advanced, life-limiting illness – the primary clinical goal that distinguishes PC from curative models.
Constitutive	Ethical framework	A strong ethical framework grounded in dignity, proportionality, and informed participation. Decisions must respect patient values and avoid futile or disproportionate interventions.
Constitutive	Family support and bereavement	An explicit commitment to supporting families throughout the illness trajectory and into bereavement – a constitutive boundary that separates PC from disease-directed care.
Constitutive	Therapeutic alliance	PC promotes a model of therapeutic alliance within interdisciplinary teams and with the patient – contrasting with paternalistic models in which decisions are unilateral and imposed.
Constitutive	Shared decision-making	Emphasizing shared decision-making, anticipatory communication, and respect for patient values as the relational grammar that constitutes PC encounters.
Constitutive	Relational patterns as identity markers	Systematized interactional patterns – recurrent configurations of behaviors, meanings, and procedures – are constitutive of PC’s disciplinary identity, not anomalies within it.
Constitutive	Clinical reflexivity and training	Competence in recognizing and navigating relational patterns constitutes a marker of clinical maturity. Integrating patterns into curricula (SPIKES, NURSE, and simulation) is a constitutive educational commitment.

This ontological and epistemological repositioning has deep implications for clinical practice and for the relational dynamics between professionals, patients, and families (Bertaud et al. 2025). PC promotes a model of therapeutic alliance within interdisciplinary teams and with the patient, emphasizing shared decision-making, anticipatory communication, and respect for patient values (Chochinov 2023). This stands in marked contrast to paternalistic models in which decisions are unilateral and imposed.

More recently, PC has also been framed as a human right (Brennan 2007; Breitbart 2008). This adds special significance to the way this clinical area is understood.

The foundational pillars and the management model remain. We have registered developments with regard to the recommendation to extend PC practices to non-cancer patients and to assure integrated care with other clinical areas as early as possible in the course of the disease (Rodrigues Mós and Reis-Pina 2025).

Drawing on an analytical autoethnographic study (Chang 2016) conducted over more than 3 decades of clinical practice with more than 12,000 patients and their families, we identified a set of characteristic relational patterns, defined as “structured and recurrent configurations of behaviors, meanings, and procedures that emerge in typical interactions.” These patterns acquire particular relevance in PC, where the clinical encounter unfolds in contexts of extreme vulnerability marked by suffering, fear, prognostic uncertainty, and anticipatory grief. Recognizing and systematizing these patterns is therefore not merely an academic exercise; it contributes to defining identity of the discipline itself and constitutes a pedagogical tool for clinical training, professional reflexivity, and quality improvement.

Although the literature describes various patterns relevant to PC, such as functional decline trajectories (Lunney et al. 2003), suffering patterns (Seng Beng et al. 2021), symptom clusters (Van Lancker et al. 2016), and communicational phenomena such as the “conspiracy of silence” (Ferreira-Nunes and Reis Pina 2026; Stiefel et al. 2024), interactional patterns are rarely addressed in a systematic and conceptually integrated manner. This work seeks to address that gap.

## Relational and cultural dynamics of identified patterns

Previous publications (Neto 2022, 2026) have detailed the patterns identified; here they are only named: “*Will he die of hunger and thirst?*,” “*The honeymoon of palliative care*,” “*The cousin from France*,” “*Please don’t take away his hope*,” “*He was doing so well! Why is he dying?*,” “*Do everything!*,” and “*He wanted to die at home*.”

Despite their diversity, these patterns share core relational and cultural dynamics and reflect universal tensions inherent to PC (hope vs. acceptance, cure vs. care, patient vs. family) (Zimmermann et al. 2021) (Table 2):

- **Persistent misconceptions about PC**

**Table 2.** Cultural elements in PC identity Table 2 long description.

Cultural values and PC identity		
Category	Core element	description
Cultural	Paradigmatic shift	Recentering clinical work from disease to person, from cure to care, and from quantity of life to its quality and meaning – a foundational cultural repositioning of medicine.
Cultural	Confronting finitude	The culture of PC is shaped by deep cultural narratives surrounding hope, duty, guilt, and mortality. Recognizing and navigating these narratives is central to practice.
Cultural	Cultural sensitivity	The meaning of patterns (e.g. “Do everything!”) varies across cultures, shaped by social representations of death, family traditions, and professional cultures. Cultural sensitivity is indispensable.
Cultural	Hope and acceptance	A foundational tension between sustaining hope and accepting irreversibility – patterns such as “Please don’t take away his hope” and “He was doing so well!” express this cultural conflict.
Cultural	PC as a human right	More recently framed as a human right, not merely a clinical service – this reflects a broader cultural and ethical recognition of access to comfort at the end of life.
Cultural	Public education	Widespread misconceptions equate PC with abandonment or imminent death. Addressing these requires anticipatory communication and broader public education.

Patterns such as “*The honeymoon of palliative care*” and “*Please don’t take away his hope*” reveal a widespread belief that PC equates to therapeutic abandonment or imminent death. When rapid symptom improvement or discharge occurs, families often express confusion, having expected decline rather than comfort. This underscores the need for anticipatory communication and public education.

- **Difficulty integrating finitude**

Patterns like “*The cousin from France*” or “*He was doing so well! Why is he dying?*” illustrate repeated questioning of prognosis and resistance to accepting irreversibility. These reactions reflect denial, guilt related to absence, anticipatory grief, and difficulty confronting finitude.

- **“Do everything!” – a request with layered meanings**

The expression rarely constitutes a literal demand for maximal intervention. Instead, it conveys distress, fear of abandonment, moral duty, or the desire to avoid future regret. Understanding these layers enables clinicians to redirect conversations toward comfort-focused and proportionate care.

- **Tension between past promises and present realities**

The pattern “*He wanted to die at home*” exemplifies how promises made without full understanding of clinical complexity can generate guilt and suffering. Clinicians often need to release families from such commitments and reorient decisions toward what best ensures patient comfort.

- **Eating and drinking at the end of life**

The pattern “*He is not eating! Will he die of hunger and thirst?*” highlights the emotional weight of reduced intake. This requires proactive conversations that normalize physiological decline and dissociate reduced intake from neglect.

## Interpretative value of the patterns

These patterns function as interpretative lenses for dynamics that are frequent yet seldom systematized. They reveal that the culture of PC is shaped not only by symptom management but also by the navigation of deep cultural narratives surrounding hope, duty, guilt, and mortality (Hira et al. 2025). Making explicit what is typically tacit enhances clinicians’ capacity to anticipate tensions and strengthen therapeutic relationships.

- **Interactional patterns as markers of disciplinary identity**

Systematizing these patterns advances the conceptualization of PC as a culturally situated clinical field. They are not anomalies but structured expressions of foundational tensions inherent to the palliative encounter: hope versus acceptance, autonomy versus dependence, care versus cure, patient versus family, and family versus team.

Their recurrence across cultures and health systems suggests that they reflect universal human dynamics activated by confrontation with serious illness and impending death. Competence in recognizing and navigating these patterns constitutes a marker of clinical maturity in PC.

- **Cultural and educational dimensions**

The cultural dimension of these patterns is essential. For example, the prevalence and meaning of “*Do everything!*” vary across cultural contexts, shaped by social representations of death, health-system structures, family traditions, and professional cultures (Bujdos et al. 2023). Cultural sensitivity is therefore indispensable.

Naming the patterns – using language drawn from everyday clinical practice – serves a pedagogical function: it enables teaching, team discussion, anticipation, and reflective supervision. Contemporary PC training increasingly incorporates structured communication models (SPIKES, NURSE, VitalTalk) (Arnold et al. 2024), simulation, and reflective approaches. Integrating interactional patterns into these curricula is a natural and necessary step. And although we do not focus here on the definition of professional identity, indirectly these scenarios can also contribute to that task.

- **Implications for research**

This article is primarily conceptual and exploratory. A subsequent phase of the study will involve empirical validation of the patterns through collaborative interviews. Future research should examine cultural variations and identify contextual factors that modulate their expression.

## Conclusion and further steps

PC is defined not only by technical expertise and symptom control but also by the relational patterns that emerge when professionals, patients, and families confront serious illness and finitude. The patterns presented here – initial idealization, the arrival of the distant relative, and cultural pressure for maximal intervention – illuminate the emotional, ethical, and cultural logics that shape PC practice.

Their systematization contributes to the construction of PC’s disciplinary identity, offering a recognizable and teachable relational grammar. More than situational diagnostic tools, these patterns invite clinical reflexivity and support clinicians in navigating relational dynamics with greater awareness, effectiveness, and compassion.

Advancing empirical validation, exploring transcultural expression, and integrating these patterns into PC training represent essential next steps. Understanding what unfolds in the clinical relationship in PC is ultimately a way of understanding what we offer to those who entrust us with their suffering at the most vulnerable moments of their lives.

## Table 1 Long description

The table lists foundational values that define PC identity, organized by category, core element, and description. All entries fall under the same category, “Constitutive,” indicating these are essential rather than optional features. The core elements include a holistic and interdisciplinary care model and a primary focus on quality of life and relief of suffering in advanced, life-limiting illness. Ethical commitments are highlighted through dignity, proportionality, informed participation, and avoiding futile or disproportionate interventions. Several elements emphasize relationships and communication: therapeutic alliance, shared decision-making, anticipatory communication, and respect for patient values, contrasted with paternalistic decision-making. Family support is treated as a defining boundary, extending care through the illness trajectory and into bereavement. The table also frames recurring relational patterns as identity markers of the discipline, not exceptions. Finally, it notes clinical reflexivity and training as constitutive, citing structured communication approaches and simulation as examples of curricular integration.

Navigate back to Table 1.

## Table 2 Long description

The table lists cultural values that shape palliative care identity, pairing each category entry with a core element description. It highlights a paradigmatic shift that recentres practice from disease and cure toward the person, care, and quality and meaning of life. It also emphasises confronting finitude, noting that narratives about hope, duty, guilt, and mortality influence how care is understood and delivered. Cultural sensitivity is presented as essential because common requests such as “Do everything!” can carry different meanings across cultures, family traditions, and professional norms. A recurring tension is described between sustaining hope and accepting irreversibility, illustrated by statements like “Please don’t take away his hope” and “He was doing so well!”. The table adds a rights-based framing of palliative care as a human right, reflecting broader ethical recognition of access to comfort at end of life. Finally, it notes that public misconceptions often equate palliative care with abandonment or imminent death, requiring anticipatory communication and public education. The entries are qualitative themes rather than measured quantities, so they summarise concepts without indicating prevalence or ranking.

Navigate back to Table 2.
